# Blue benzoquinone from scorpion venom shows bactericidal activity against drug-resistant strains of the priority pathogen *Acinetobacter baumannii*

**DOI:** 10.1038/s41429-025-00809-8

**Published:** 2025-02-18

**Authors:** Ramses Gallegos-Monterrosa, Jimena I. Cid-Uribe, Gustavo Delgado-Prudencio, Deyanira Pérez-Morales, María M. Banda, Alexis Téllez-Galván, Edson N. Carcamo-Noriega, Ulises Garza-Ramos, Richard N. Zare, Lourival D. Possani, Víctor H. Bustamante

**Affiliations:** 1 Departamento de Microbiología Molecular, Instituto de Biotecnología, Universidad Nacional Autónoma de México, C.P. 62210 Cuernavaca, Morelos México; 2 Departamento de Medicina Molecular y Bioprocesos, Instituto de Biotecnología, Universidad Nacional Autónoma de México, C.P. 62210 Cuernavaca, Morelos México; 3 Instituto Nacional de Salud Pública, Centro de Investigación Sobre Enfermedades Infecciosas, Laboratorio de Resistencia Bacteriana, C. P. 62210 Cuernavaca, Morelos México; 4 Department of Chemistry, Stanford University, Stanford, CA 94305 USA; 5 Present Address: Programa de Biología de Sistemas, Centro de Ciencias Genómicas, Universidad Nacional Autónoma de México, C.P. 62210 Cuernavaca, Morelos México; 6 Present Address: Programa de Microbiología Genómica, Centro de Ciencias Genómicas, Universidad Nacional Autónoma de México, C.P. 62210 Cuernavaca, Morelos México

**Keywords:** Antibiotics, Bacteria

## Abstract

Antibiotic-resistant bacteria pose a significant global health threat, particularly pathogens resistant to last-resort antibiotics, such as those listed as priority pathogens by the World Health Organization. Addressing this challenge requires the development of novel antimicrobial agents. Previously, we identified a blue 1,4-benzoquinone isolated from the venom of the Mexican scorpion *Diplocentrus melici* as a potent antimicrobial compound effective against *Staphylococcus aureus* and *Mycobacterium tuberculosis*. Moreover, we devised a cost-effective synthetic route for its production. In this study, we demonstrate that the blue benzoquinone exhibits antibacterial activity against additional pathogens, including the priority pathogen *Acinetobacter baumannii*. Notably, the compound effectively killed clinical strains of *A. baumannii* resistant to multiple antibiotics, including carbapenem and colistin. Furthermore*, A. baumannii* did not develop resistance to the benzoquinone even after multiple growth cycles under sub-inhibitory concentrations, unlike the tested antibiotics. These findings underscore the potential of this blue benzoquinone as a lead compound for the development of a new class of antibiotics targeting multidrug-resistant bacteria.

## Introduction

The rise and spread of antimicrobial resistance (AMR) among pathogenic bacteria represent a major health crisis. It was estimated that in 2019 there were worldwide approximately 5 million human deaths associated with, and 1.27 million human deaths directly caused by antimicrobial-resistant bacteria [1]. Even more worrisome, if current trends continue, by 2050 the number of AMR-related deaths could reach 10 million annually, making it the first cause of death for humans [2].

Bacteria can develop antibiotic resistance (AR) through different molecular mechanisms, including among others the expression of enzymes that inactivate antibiotics, the expression of efflux pumps that expel antibiotics out of the cells, and DNA mutations that lead to the expression of target molecules that are not recognized by antibiotics [3, 4]. Additionally, bacteria can transmit genetic determinants for AR to other bacteria through horizontal gene transfer events mediated by distinct mechanisms, which permit rapid spread of AR [3, 5].

The development of drug-resistance is promoted when bacteria are exposed to sub-inhibitory concentrations of antimicrobials [6, 7]. Worryingly, bacteria can accumulate genetic determinants for AR resulting in multi-drug resistant (MDR) bacteria (resistant to three or more antimicrobials of different categories), extensively drug-resistant (XDR) bacteria (resistant to all but one or two antibiotic categories), and pan-drug resistant (PDR) bacteria (resistant to all antimicrobial categories) [8]. Drug-resistant bacteria cause infections that are more difficult to treat and have a higher probability of treatment failure; drug-resistant bacteria are correlated with enhanced morbidity and mortality [9, 10]. In 2015, the World Health Organization (WHO) published a global action plan to control AMR, which includes the development of new antimicrobial compounds as one major objective [11]. Additionally, in 2017 the WHO published a list of drug-resistant bacteria denominated priority pathogens for which the development of new antimicrobials is urgently needed. This list was actualized by the WHO in 2024; both lists include carbapenem-resistant *Acinetobacter baumannii* in the group of critical priority pathogens, which represent the highest threat to public health [12, 13].

*A. baumannii* is a gram-negative bacterium that has emerged in recent decades as one of the top opportunistic pathogens in hospital settings; in addition to colonizing patients it can persist in different surfaces by forming biofilms, which allows its survival when exposed to disinfectants [14]. This bacterium can cause a variety of diseases such as skin and urinary tract infections, meningitis, and blood-stream infections; notably, *A. baumannii* is a major causative agent of ventilator-associated pneumonia in intensive-care unit patients, where drug-resistant strains of this bacterium can lead to high-mortality infections [15–17]. Indeed, the rise and spread of MDR and XDR strains of *A. baumannii* have become a serious problem in healthcare settings, where strains resistant to last-resort antibiotics such as carbapenems and colistin have become increasingly common [17–19]. Two main mechanisms for colistin resistance have been described in *A. baumannii*: (1) loss of the lipopolysaccharide (LPS), the target molecule of colistin, by mutations that inactivate the genes of the lipid A biosynthetic pathway (*lpxA*, *lpxC*, and *lpxD*); (2) modification of the LPS by mutations in the genes encoding the PmrAB two-component system, resulting in the overexpression of the PmrC enzyme that adds phosphoethanolamine moieties to lipid A [19]. Carbapenem resistance in *A. baumannii* is mostly mediated by the production of OXA-type carbapenemase enzymes that hydrolyze these antibiotics [18]. In 2019 over 132,000 deaths worldwide were directly attributable to infections caused by drug-resistant strains of *A. baumannii*, making it the 4th most lethal bacterial pathogen for that year [1].

During the 20th century, soil microorganisms, particularly bacteria of the Actinomycetota phylum, served as a rich source for identifying antimicrobial compounds [20]; however, in the last years no new classes of antimicrobials have been discovered from this source [21]. Therefore, alternative niches for searching novel antimicrobial compounds have been investigated, such as plants, fungi, animals, and microbes from other environments [22, 23]. Interestingly, venoms from animals such as snakes, bees, and scorpions are being explored as sources of molecules with antimicrobial, anti-inflammatory, or other biological activities [24, 25].

Previously, we reported a blue 1,4-benzoquinone derivative purified from venom of the *Diplocentrus melici* scorpion that displays interesting bioactive properties such as antimicrobial activity against *Staphylococcus aureus* and *Mycobacterium tuberculosis*, as well as antineoplastic activity against various carcinoma cell lines [26]. In this study we found that this benzoquinone presents antimicrobial activity against other pathogenic bacteria; particularly, it shows bactericidal activity against MDR strains of *A. baumannii*, including strains resistant to the last-resort antibiotics carbapenems and colistin. Interestingly, *A. baumannii* was not able to develop resistance to this benzoquinone after multiple culture cycles with exposition to sub-inhibitory concentrations of this compound. Thus, our study reveals additional properties of the blue 1,4-benzoquinone that increase its potential as a lead compound for the development of a new class of antibiotics.

## Materials and methods

### Bacterial strains and growth media

Table 1 lists all bacterial strains used in this study. Bacteria were regularly grown in Mueller-Hinton (MH) broth (Difco; beef extract 2 g, casein acid digest 17.5 g, and soluble starch 1.5 g, in 1 L distilled water) at 37 °C with 200 rpm shaking. MH or modified M9 minimal medium (Na_2_HPO_4_ 12.8 g, KH_2_PO_4_ 3 g, NaCl 0.5 g, NH_4_Cl 1 g, glucose 4 g, MgSO_4_ 0.2407 g, CaCl_2_ 11 mg, and casamino acids 1 g, in 1 L distilled water) [27] were used to analyze bacterial growth kinetics. When solid plates were required, media were supplemented with 15 g L^−1^ agar.

**Table 1:** Bacterial strains used in this study

Bacteria	Description	Source
*Acinetobacter baumannii*		
ATCC 17978	Reference strain	ATCC
7024	Producer of OXA β-lactamases; IPM^R^, MEM^R^	[47]
8200	Producer of OXA β-lactamases; IPM^R^, MEM^R^	[47]
8407	Producer of OXA β-lactamases; IPM^R^, MEM^R^, CL^R^	[48]
8509	Producer of OXA β-lactamases; IPM^R^, MEM^R^, CL^R^	[48]
10324	Producer of OXA β-lactamases; MEM^R^	[47]
17800	Producer of OXA β-lactamases; TZP^R^, FEP^R^, CAZ^R^, IPM^R^, MEM^R^, CIP^R^	Garza-Ramos U., unpublished
17849	Producer of OXA β-lactamases; TZP^R^, AMK^R^, FEP^R^, CAZ^R^, IPM^R^, MEM^R^, CIP^R^	Garza-Ramos U., unpublished
18474	Producer of OXA β-lactamases; TZP^R^, AMK^R^, FEP^R^, CAZ^R^, IPM^R^, MEM^R^, CIP^R^, CL^R^	Garza-Ramos U., unpublished
18477	Producer of OXA β-lactamases; TZP^R^, FEP^R^, CAZ^R^, IPM^R^, MEM^R^, CIP^R^	Garza-Ramos U., unpublished
18555	Producer of OXA β-lactamases; AMK^R^, TZP^R^, FEP^R^, CAZ^R^, IPM^R^, MEM^R^, CIP^R^, CL^R^	Garza-Ramos U., unpublished
19604	Producer of OXA β-lactamases; AMK^R^, TZP^R^, FEP^R^, CAZ^R^, IPM^R^, MEM^R^, CIP^R^, TGC^R^, CL^R^	Garza-Ramos U., unpublished
Other species		
*Staphylococcus aureus* ATCC 29213	Reference strain	ATCC
*Pseudomonas aeruginosa* ATCC 27853	Reference strain	ATCC
*Enterococcus faecalis* ATCC 24212	Reference strain	ATCC
*Klebsiella pneumoniae* ATCC 700603	Reference strain	ATCC
*Salmonella enterica* serovar Typhimurium SL1344	Reference strain	[49]

ATCC: American Type Culture Collection. Antibiotics: amikacin (AMK), piperacillin-tazobactam (TZP), ceftazidime (CAZ), cefepime (FEP), imipenem (IPM), meropenem (MEM), ciprofloxacin (CIP), streptomycin (STS), tigecycline (TGC), and colistin (CL). ^R^ Resistance

### Chemical synthesis of the blue 1,4-benzoquinone

The blue 1,4-benzoquinone was synthetized as previously described [26]. Briefly, 1,4-dimethoxy-2,3-dibromobenzene was oxidized with ceric ammonium nitrate to provide 2,3-dibromo-1,4-benzoquinone; a Diels−Alder cycloaddition between this last compound and cyclopentadiene was performed, and the bromides of the resulting product were replaced with thiomethoxy groups using the protocol described by Ferreire et al. [28]. The obtained compound was heated with NaHCO_3_ in a mixture of tetrahydrofuran/MeOH/H_2_O to produce the enol tautomer. Oxidation with Fe_2_(SO_4_)_3_ in acidic MeOH produced a 1,4-benzoquinone. Finally, a *retro*-Diels−Alder reaction was carried out to yield the blue 1,4-benzoquinone.

### Preparation of benzoquinone solutions

The blue 1,4-benzoquinone was dissolved in 50% ethanol, and its concentration was determined using the extinction coefficient of 9401.7 M^−1^ cm^−1^ at 325 nm as described previously [26]. This benzoquinone stock solution was then diluted with water or culture medium as required for the different assays. The maximum ethanol concentration that remained in the samples with the benzoquinone was 3%.

### Bacterial growth inhibition in liquid medium

The antimicrobial activity of the blue 1,4-benzoquinone in liquid medium was examined under shaken or static conditions. To obtain growth kinetics in shaken conditions, bacteria were grown in MH broth up to an optical density at 600 nm (O.D._600_) of 0.6 (∼1 × 10^8^ CFU mL^−1^; equivalent to 0.5 McFarland). Then, the bacterial cultures were diluted 1:100 using fresh MH broth and 200 µL of these bacterial suspensions containing or not the benzoquinone at 10, 20, 30 or 40 µg mL^−1^ were incubated in a 96-well microplate for 18 h. Ethanol at a final concentration of 3% and gentamicin at 4 µg mL^−1^ were used as positive and negative growth controls, respectively. To examine the effect of multi-dose benzoquinone exposure, 200 µL of bacterial suspensions with benzoquinone at 30 µg mL^−1^ or 3% ethanol were prepared in a 96-well microplate as described above and incubated for 40 h. Throughout the incubation period, every 8 h, 20 µL of bacterial suspension were removed from the wells, and 20 µL of either benzoquinone solution at 300 µg mL^−1^ or ethanol were added.

Assays in M9 minimal medium were performed as previously described for cultures in MH broth, with incubation periods of 24 or 40 h. Bovine serum albumin (BSA) at 2 mg mL^−1^ was added when required.

Bacterial growth kinetics were obtained by measuring O.D._600_ every 30 min with incubation at 37 °C and shaking of 237 rpm.

For assays in static conditions, bacterial cultures containing blue 1,4-benzoquinone at 10 or 100 µg mL^−1^ were initiated as described above for growth kinetics assays, and then were incubated for 18 h in a static incubator at 37 °C. Bacterial cultures containing 10 µg mL^−1^ of meropenem and cultures without any antibiotics were assessed as controls.

The minimum inhibitory concentration (MIC) of the blue 1,4-benzoquinone, gentamicin, and meropenem, against *A. baumannii* strains was determined under static conditions using the broth microdilution method according to the Clinical and Laboratory Standards Institute (CLSI) guidelines [29]. Serial 2-fold dilutions of the antimicrobials were prepared using cation-adjusted MH broth (CAMHB), with the highest antimicrobial concentration being 256 µg mL^−1^. Then, 100 µL of these dilutions were loaded on a 96-well microplate in triplicate and inoculated with ∼5 × 10^4^ CFU mL^−1^ of the respective bacterial strain. Bacterial cultures without any antimicrobials and CAMHB without bacterial inoculation were assessed as controls. The plates were incubated at 37 °C for 16–24 h and bacterial growth was determined.

All O.D._600_ measurements were done in an Epoch2 microplate reader (BioTek).

### Bacterial kill rates

Bacterial kill rates were examined as previously described [30]. Shortly, bacterial suspensions in phosphate-buffered saline solution (PBS) (1.8 mM NaH_2_PO_4_, 10 mM Na_2_HPO_4_, 2.7 mM KCl and 137 mM NaCl, pH 7.4) were incubated with or without benzoquinone during indicated times (0 min, 30 min, 1 h, 2 h, and 3 h) and colony forming units (CFUs) were recorded by spotting 10 µL of serial dilutions of bacterial suspension on MH agar plates.

### Development of antimicrobial resistance

Development of antimicrobial resistance by *A. baumannii* ATCC 17978 was evaluated using a multi-step resistance selection experiment as described previously [31]. On day 1 of the experiment, 100 μL of fresh MH broth containing the blue 1,4-benzoquinone or antibiotics (ciprofloxacin or gentamicin) at concentrations 0.5X (initial sub-inhibitory concentration), 1X, 2X, or 4X of their respective MIC were added in duplicate into 96-well plates. Then, 10 μL of a culture of *A. baumannii* ATCC 17978 grown up to an O.D._600_ of 0.6 were inoculated in each well. After 24 h of incubation at 37 °C under static conditions, the growth of the bacterial cultures was determined by measuring the O.D._600_. Next, 10 μL of each bacterial culture from the highest concentration of antimicrobial showing growth were used as the inoculum for a subsequent culture containing antimicrobial concentrations 0.5X, 1X, 2X, or 4X of the MIC showed in the previous culture. Similar 24 h cycle-cultures were repeated over a total of 35 days. Results are reported as the increase in MIC values detected through the time assessed. According to the CLSI, the MICs of ciprofloxacin and gentamicin for *A. baumannii* are 1 and 4 µg mL^−1^, respectively [32].

### Analysis of the benzoquinone absorption spectrum

The absorption spectrum of the blue 1,4-benzoquinone at 30 µg mL^−1^ was determined in cell-free MH or M9 liquid media, or in a 3% ethanol solution, over a wavelength range of 270–470 nm. The assays in culture media were performed in microplates containing 200 µL of medium, incubated at 37 °C for 24 h. The assays in ethanol solution were performed in tubes containing 500 µL of ethanol solution, incubated at 37 °C for 72 h. Absorption measurements were taken every hour for assays in culture media and every 24 h for assays in ethanol solution, using an Epoch2 microplate reader (BioTek).

### Statistical analysis

Statistical significance was determined by two-tailed Student’s *t* test, using GraphPad Prism v8.0.1 software (GraphPad Inc., San Diego, CA) and assuming dissimilar variance among groups. *P* values ≤ 0.05 were considered significant.

## Results

### The blue 1,4-benzoquinone shows antimicrobial activity against different pathogenic bacteria

**Fig. 1:**
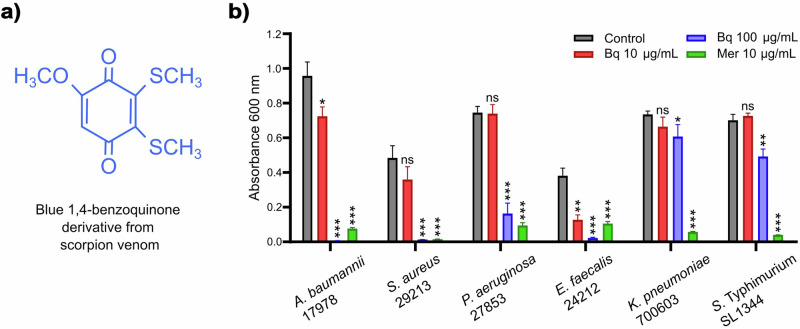
Structure and antibacterial activity of the blue 1,4-benzoquinone. **a** Structure of the blue 1,4-benzoquinone derivative obtained from venom of *Diplocentrus melici* [26]. **b** Antibacterial activity was tested by growing bacteria in MH broth containing 10 or 100 µg mL^−1^ of the benzoquinone (Bq) or 10 µg mL^−1^ of meropenem (Mer). Cultures lacking antimicrobials were tested as controls. Bacterial cultures were grown for 18 h at 37 °C under static conditions. Data represent the average of three independent experiments done in triplicate; error bars represent standard error of the mean. For each bacterial species, data statistically different with respect to its control are indicated: ns not significant; * *P* ≤ 0.05; ** *P* ≤ 0.005; *** *P* ≤ 0.0009

To determine whether the blue 1,4-benzoquinone (Fig. 1a) exhibits antimicrobial activity against other clinically relevant pathogenic bacteria, in addition to its activity against *M. tuberculosis* and *S. aureus* that we reported previously [26], the growth-inhibitory effect of this compound at concentrations of 10 and 100 µg mL^−1^ was tested on bacterial cultures grown in MH broth. The benzoquinone at 100 µg mL^−1^ inhibited the growth of the *A. baumannii* ATCC 17978, *S. aureus* ATCC 29213, *Enterococcus faecalis* ATCC 24212, and *Pseudomonas aeruginosa* ATCC 27853 strains, whereas at 10 µg mL^−1^ it only significantly inhibited the growth of the *A. baumannii* ATCC 17978 and *E. faecalis* ATCC 24212 strains (Fig. 1b). As expected, meropenem at 10 µg mL^−1^, tested as a control, inhibited the growth of all the assessed bacterial strains (Fig. 1b).

These results show that the blue 1,4-benzoquinone has antimicrobial activity against both gram-positive and gram-negative pathogenic bacteria.

### The blue 1,4-benzoquinone inhibits the growth of *A. baumannii* in a dose-dependent way

**Fig. 2:**
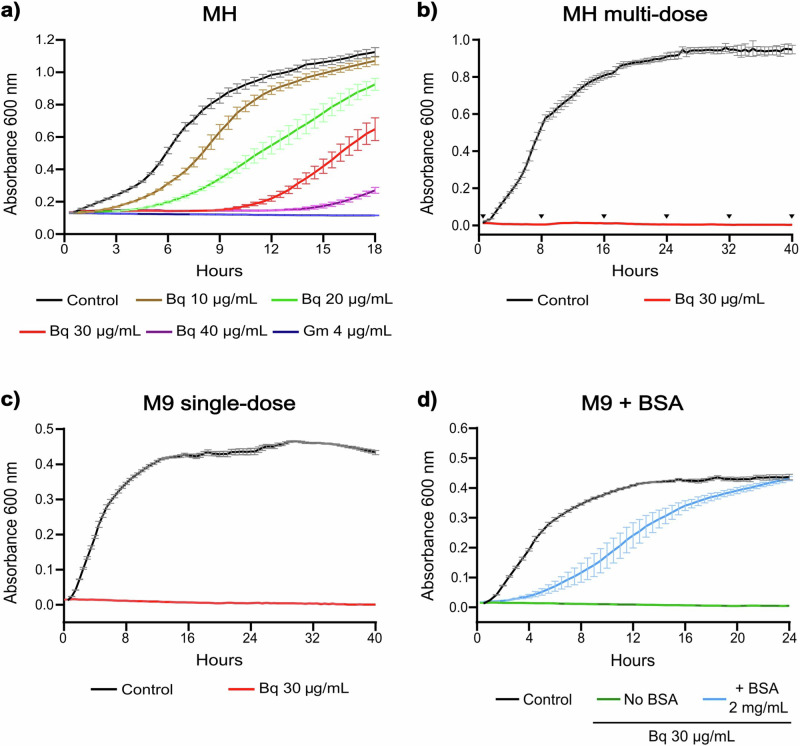
The blue 1,4-benzoquinone inhibits the growth of *A. baumannii*. **a** Growth kinetics of the *A. baumannii* ATCC 17978 strain in MH broth with different concentrations of the benzoquinone (Bq): 10, 20, 30 or 40 µg mL^−1^. Gentamicin (Gm) at 4 µg mL^−1^ was tested as a positive control for growth inhibition. **b** Growth kinetics of the *A. baumannii* ATCC 17978 strain in MH broth with doses of Bq at 30 μg mL^−1^ applied every 8 h (indicated in the figure with black arrowheads). **c** Growth kinetics of the *A. baumannii* ATCC 17978 strain in M9 broth with a single-dose of Bq at 30 μg mL^−1^. **d** Growth kinetics of the *A. baumannii* 17978 strain in M9 broth with Bq at 30 μg mL^−1^ and bovine serum albumin (BSA) at 2 mg mL^−1^. In all panels, the control lacks benzoquinone. Data represent the average of three independent experiments done in triplicate; error bars represent standard error of the mean

Because *A. baumannii* has been classified by the WHO as a critical priority pathogen for the development of new antimicrobials [12], we decided to focus the remaining of our investigation on this bacterium. We examined the effect of increasing concentrations of the blue 1,4-benzoquinone on growth kinetics of *A. baumannii* ATCC 17978 in MH broth. The benzoquinone inhibited the growth of *A. baumannii* ATCC 17978 in a dose-dependent manner at concentrations of 10, 20, 30, and 40 µg mL^−1^ (Fig. 2a). At concentrations of 30 and 40 µg mL^−1^ the blue 1,4-benzoquinone inhibited the bacterial growth during 10 and 14 h, respectively; after these times, bacterial re-growth was observed (Fig. 2a). As expected, gentamicin prevented the growth of *A. baumannii* ATCC 17978 during the full time assessed (Fig. 2a). These results prompted us to wonder whether repeated applications of benzoquinone could inhibit the growth of *A. baumannii* ATCC 17978 without allowing bacterial re-growth. To investigate this possibility, growth kinetics of *A. baumannii* ATCC 17978 were determined in MH broth lacking benzoquinone or with additions of benzoquinone at 30 µg mL^−1^ every 8 h. With multi-dose of the benzoquinone the growth of *A. baumannii* ATCC 17978 remained inhibited during the 40 h assessed (Fig. 2b). As a possibility to explain the bacterial re-growth after a time with a single dose of benzoquinone, we thought that the complex components of the MH rich-medium could be affecting the antimicrobial activity of this compound. Consistent with this idea, pre-incubation of the benzoquinone for 12 h in MH broth, without bacteria, accelerated the appearance of re-growth of *A. baumannii* ATCC 17978 in the presence of this compound (Fig. S1). Then, we examined the effect of the benzoquinone on the growth kinetic of *A. baumannii* ATCC 17978 in M9 minimal medium. Notably, a single-dose of benzoquinone at 30 µg mL^−1^ completely inhibited the growth of *A. baumannii* ATCC 17978 in M9 minimal medium during the 40 h assessed (Fig. 2c).

Together, these results indicate that the blue 1,4-benzoquinone shows dose-dependent antimicrobial activity against *A. baumannii*. Additionally, these results suggest that components of the MH rich-medium affect in some way the antimicrobial activity of this benzoquinone.

### The blue 1,4-benzoquinone shows bactericidal activity against drug-resistant strains of *A. baumannii*

**Fig. 3:**
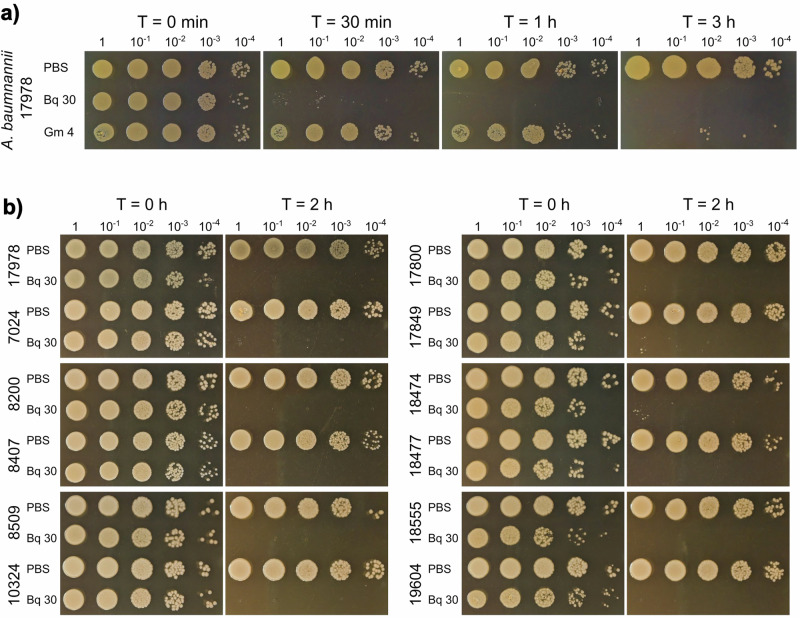
Bactericidal activity of the blue 1,4-benzoquinone against *A. baumannii*. **a** Kill rates of the benzoquinone against the *A. baumannii* ATCC 17978 strain. Bacterial suspensions in PBS were exposed to the benzoquinone at 30 µg mL^−1^ (Bq 30) during indicated times with incubation at 37 °C. Gentamicin at 4 µg mL^−1^ (Gm 4) was used as a positive control for bactericidal activity. Bacterial suspensions not exposed to the benzoquinone are indicated as PBS. CFUs were recorded by spotting serially diluted samples of the bacterial suspensions on MH agar plates at different exposition times (0 min, 30 min, 1 h, 2 h, and 3 h). **b** Kill rates of the benzoquinone against MDR strains of *A. baumannii*. Bacterial suspensions in PBS were exposed to the benzoquinone at 30 µg mL^−1^ (Bq 30) during 2 h with incubation at 37 °C. Bacterial suspensions not exposed to the benzoquinone are indicated as PBS. CFUs were recorded by spotting serially diluted samples of the bacterial suspensions on MH agar plates at the start and the end of the exposure period. Representative images from three independent experiments are shown

To determine whether the antimicrobial activity showed by the blue 1,4-benzoquinone against *A. baumannii* is caused by a bacteriostatic or bactericidal effect, bacterial survival after exposition to benzoquinone was analyzed. Suspensions containing ≈ 3 × 10^7^ bacteria per mL of *A. baumannii* ATCC 17978 in PBS were exposed to the benzoquinone at 30 µg mL^−1^ during different times. Then, CFUs were recorded by spotting serially diluted samples onto MH agar plates. The bactericidal antibiotic gentamicin at 4 µg mL^−1^ (MIC value) was assessed as a positive control. As expected, CFUs from the suspensions lacking the benzoquinone remained constant through the times tested (Fig. 3a). In contrast, in the suspension containing the benzoquinone, no CFUs were detected after 30 min of exposition (Fig. 3a), indicating that the benzoquinone has a bactericidal effect on *A. baumannii* ATCC 17978. Interestingly, the benzoquinone killed *A. baumannii* ATCC 17978 even faster than gentamicin at its respective MIC: gentamicin eradicated bacteria after 3 h of exposition (Fig. 3a).

Next, we tested the bactericidal activity of the benzoquinone against 11 clinical isolates of *A. baumannii* resistant to multiple antibiotics, including carbapenem and colistin (see Table 1). Notably, the benzoquinone was able to kill all tested *A. baumannii* clinical strains after 2 h of exposition, indicating that it exhibits bactericidal activity against *A. baumannii* even in the presence of mechanisms providing resistance to different antibiotics, including those for last-resource antibiotics (Fig. 3b). In agreement with these results, MIC determination by the 2-fold microdilution method showed that the benzoquinone inhibits the growth of the 12 *A. baumannii* strains used in this study at concentrations of 32 or 64 µg mL^−1^ (Table S1). As expected, the viability of eight *A. baumannii* clinical isolates tested was not affected by the antibiotics gentamicin and meropenem, respectively, after 4 h of exposition (Fig. S2), supporting that these clinical isolates are resistant to both gentamicin and meropenem. Moreover, MIC determination confirmed that the 11 *A. baumannii* clinical isolates, but not the *A. baumannii* ATCC 17978 strain, are resistant to both gentamicin and meropenem (Table S1).

In summary, our results show that the blue 1,4-benzoquinone possesses bactericidal activity against antibiotic-resistant strains of the priority pathogen *A. baumannii*.

### Continuous exposure to the blue 1,4-benzoquinone did not result in the development of resistance in *A. baumannii*

**Fig. 4:**
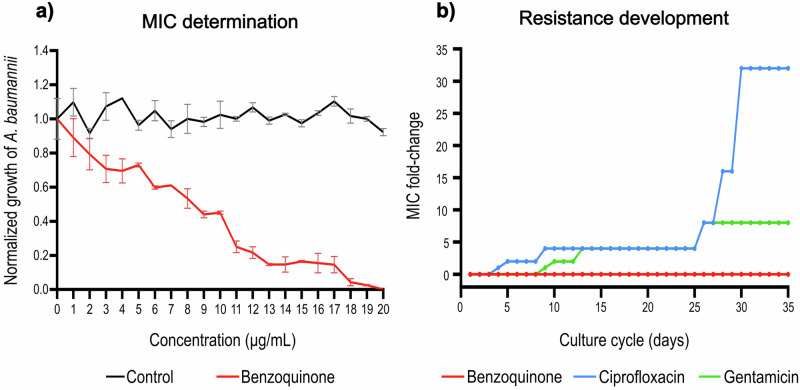
*A. baumannii* did not develop resistance to the blue 1,4-benzoquinone after continuous exposure. **a** Determination of the minimal inhibitory concentration (MIC) of the benzoquinone against the *A. baumannii* ATCC 17978 strain. Bacteria were grown in MH broth without (control) or with different concentrations of the benzoquinone under static conditions. Data represent the average of three independent experiments done in triplicate; error bars represent standard error of the mean. **b** Serial culture cycles of the *A. baumannii* 17978 strain in MH broth with different concentrations of the benzoquinone, ciprofloxacin or gentamicin (0.5X, 1X, 2X, and 4X of the MIC). Bacterial cultures were incubated in static conditions. Data show the increase in the MIC of each compound against *A. baumannii* ATCC 17978 throughout the growth cycles

Low propensity for inducing resistance is a desirable property for an optimal antibiotic. Thus, we investigated whether the blue 1,4-benzoquinone has this property. Firstly, we determined more precisely the MIC of the benzoquinone against *A. baumannii* ATCC 17978 in static conditions, with increases of 1 µg mL^−1^, which was found to be 20 µg mL^−1^ (Fig. 4a). Then, a multi-step resistance selection experiment [31] was conducted under static conditions to determine whether the continuous exposure to a sub-inhibitory concentration (0.5× of the 20 µg mL^−1^ MIC) of the benzoquinone leads to the development of resistance in *A. baumannii* ATCC 17978. Two different classes of antibiotics were used as controls in these assays at 0.5× of their respective MIC: ciprofloxacin (DNA synthesis inhibitor) and gentamicin (protein synthesis inhibitor). Notably, development of resistance to the benzoquinone was not observed in *A. baumannii* ATCC 17978 during uninterrupted 35 days of sequential culture cycles (Fig. 4b). In contrast, *A. baumannii* ATCC 17978 developed resistances to the two tested antibiotics after a few culture cycles, reaching at the end of the assay increases in the MICs of 32- and 8-fold for ciprofloxacin and gentamicin, respectively (Fig. 4b).

These results indicate that *A. baumannii* does not exhibit the propensity to develop resistance against the blue 1,4-benzoquinone.

### The antibacterial activity of the blue 1,4-benzoquinone is affected by the presence of proteins in culture media

As described above, growth-inhibition of the *A. baumannii* 17978 strain by the blue 1,4-benzoquinone was lost overtime in the MH rich-medium but not in the M9 minimal-medium, and also by pre-incubation of the benzoquinone in the MH rich-medium without bacteria (Figs. 2a, c, and S1). These results suggest that the benzoquinone is affected in some way by components of the MH medium. To investigate this, benzoquinone at 30 µg mL^−1^ was mixed with cell-free MH or M9 liquid medium and its absorption spectrum in the range of 270–470 nm was measured through time. In MH broth, the benzoquinone showed shifts in the wavelength of its maximum absorbance peak, as well as an increase in its absorbance through time, starting at 6 h (Fig. S3a). In contrast, the absorption spectrum of the benzoquinone in M9 broth showed moderate changes through time (Fig. S3b). Important to note, the absorption spectrum of the benzoquinone remained without changes during 72 h in 3% ethanol solution (Fig. S3d), the final concentration of ethanol present in the growth media containing benzoquinone. These results suggest that the benzoquinone interacts with component(s) present in the MH broth. A major difference between the MH and M9 media is a high content of proteins in the MH medium. Thus, we decided to investigate if the addition of bovine serum albumin (BSA) to the M9 broth affects the absorption spectrum and/or the antibacterial activity of the benzoquinone. The presence of BSA slightly modified the benzoquinone´s absorption spectra through time (Fig. S3c) and reduced the antibacterial activity of the benzoquinone against *A. baumannii* (Fig. 2d) in M9 broth. In contrast, the presence of BSA did not alter the antimicrobial activity of the antibiotics gentamicin, meropenem, or tetracycline in the M9 medium; bacterial re-growth was observed in the medium containing tetracycline, but it was independent of the BSA (Fig. S4).

Together, our results suggest that the presence of protein in culture media affects the bioavailability of the blue benzoquinone and thus its antibacterial activity.

## Discussion

Previously we identified the blue 1,4-benzoquinone as a compound with antimicrobial activity against *M. tuberculosis* and *S. aureus* [26]. In this study, we found that this benzoquinone also shows antimicrobial activity against the pathogenic bacteria *A. baumannii*, *E. faecalis*, and *P. aeruginosa* (Fig. 1b). Notably, the benzoquinone had bactericidal activity against MDR strains of *A. baumannii*, including strains resistant to carbapenems and/or colistin (Fig. 3). Carbapenems are a class of wide-spectrum β-lactam antibiotics that are considered to be the primary drug to treat patients with severe hospital-acquired bacterial infections, including those by *A. baumannii* [18]. Worryingly, the isolation of carbapenem-resistant strains of *A. baumannii* from hospital environments has increased globally in recent years, from 1% in 2003 to 58% in 2008 in some North American hospitals [33]; while more than 70% of pathogenic *Acinetobacter* isolates in Southern and Eastern Europe were carbapenem-resistant between 2013 and 2017 [34]. In 2019, 40 to ≥ 80% of the isolates of *A. baumannii* were estimated to be carbapenem-resistant in many countries around the world [1]. The WHO has declared carbapenem-resistant *A. baumannii* as a pathogen of critical priority for the development of new antimicrobials [12]. On the other hand, colistin (polymyxin E) is a peptidic bactericidal antibiotic with limited applications in healthcare owing to its nephrotoxicity and complicated dossing; nevertheless, it is considered as a last-resort antibiotic to treat severe MDR *A. baumannii* infections [19, 35]. In 2020, a meta-analysis of 150 independent studies found that colistin resistance in *A. baumannii* isolates was as high as 12 and 17% in some regions of Southeast Asia and the Eastern Mediterranean regions, respectively [36]. Thus, the ability of the blue 1,4-benzoquinone to eradicate *A. baumannii* clinical strains resistant to carbapenems and/or colistin is highly relevant.

In addition to AR, the failure of antibiotic treatment during infection is associated with the development of a metabolically inactive subpopulation of bacterial cells that do not display growth; because most antibiotic classes act on metabolically active bacteria, these non-growing cells are not affected by those antibiotics [37, 38]. The identification of antimicrobials that are able to kill non-growing bacterial cells has thus become an important issue for combating recalcitrant or chronic bacterial infections [39]. The blue 1,4-benzoquinone was able to kill *A. baumannii* in PBS solution (Fig. 3), which does not support bacterial growth. Thus, our results reveal the antimicrobial activity on non-growing bacteria as an additional relevant antibiotic property of the blue 1,4-benzoquinone.

We show that *A. baumannii* remains sensitive to the blue 1,4-benzoquinone after 35 sequential growth cycles of exposure to sub-inhibitory concentrations of this compound; in contrast, *A. baumannii* developed resistance toward the ciprofloxacin and gentamicin antibiotics (Fig. 4b). These results suggest that the blue 1,4-benzoquinone has low propensity for resistance development by *A. baumannii*, which is also a remarkable property for an optimal antibiotic.

Various benzoquinone derivatives have antioxidant, anti-inflammatory, or antimicrobial properties; some of them are already used for the synthesis of heterocyclic drugs [40]. It has been previously shown that 1,4-benzoquinones are highly reactive toward macromolecules such as proteins [41]. For instance, 1,4-benzoquinones bind to albumin and form albumin adducts [42]; likewise, they can bind to the thiol groups of microtubule proteins [43]. We speculate that the blue 1,4-benzoquinone studied here exerts its antibacterial activity by interacting with membrane proteins, probably causing disruption of the bacterial envelope, which remains to be determined. The bacterial re-growth observed in the MH broth could be caused by interaction of the blue 1,4-benzoquinone with proteins from this rich medium (Figs. S1 and S3), which could diminish the bioavailability of the benzoquinone to reach its bacterial targets. Supporting this hypothesis, we show that the presence of BSA causes bacterial re-growth in M9 broth containing a growth-inhibitory concentration of the blue 1,4-benzoquinone (Fig. 2d). Similarly to the observed phenomenon for the blue benzoquinone, results from a previous study show re-growth of *Escherichia coli* in MH broth containing 1× MIC of the antibiotic levofloxacin [44].

We reach the conclusion that the blue 1,4-benzoquinone displays attractive properties as a lead molecule for the development of a new antibiotic. In addition to the properties described in this study, we previously reported that the blue 1,4-benzoquinone shows in vitro antibacterial activity against *M*. *tuberculosis* and *S. aureus*, as well as therapeutic effect for the *M*. *tuberculosis* infection in mice [26]. Furthermore, we previously found that this benzoquinone does not induce hemolysis of human erythrocytes and shows cytotoxic activity against different neoplastic cell lines; however, it also shows considerable cytotoxic activity against human peripheral blood mononuclear cells [26]. The cytotoxic effect and probably the antibacterial activity of the blue benzoquinone could be caused by interactions with cellular proteins and/or by induction of reactive oxygen species production, mechanisms reported for several other quinones [45]. Pharmacological strategies such as chemical modification and encapsulation in nanoparticles or liposomes have been successfully applied to improve desirable activities and to reduce harmful effects of benzoquinones compounds [40, 46]. Currently, we are following these and other strategies to further characterize the blue 1,4-benzoquinone as a compound for possible clinical application.

## Supplementary information

Supplementary Material
