# Supplementary material for: Blue benzoquinone from scorpion venom shows bactericidal activity against drug-resistant strains of the priority pathogen Acinetobacter baumannii
Source: J Antibiot (Tokyo). 2025 Feb 18;78(4):235–45. doi: 10.1038/s41429-025-00809-8 (PMC11946886; doi:10.1038/s41429-025-00809-8)
Supplement: Supplementary file 1 — Supplementary Material [file 41429_2025_809_MOESM1_ESM.docx]

**A blue benzoquinone from scorpion venom shows bactericidal activity against drug-resistant strains of the priority pathogen *Acinetobacter baumannii***

**SUPPLEMENTARY MATERIAL**

**Table S1.** Minimum inhibitory concentrations of antimicrobials against *A. baumannii* strains used in this study.

| **Strain** | **Benzoquinone**  **(µg mL^-1^)** | **Gentamicin**  **(µg mL^-1^)** | **Meropenem**  **(µg mL^-1^)** |
| --- | --- | --- | --- |
| 17978 | 32 | 4 (S) | 2 (S) |
| 7024 | 64 | 128 (R) | 128 (R) |
| 8200 | 32 | 16 (R) | 256 (R) |
| 8407 | 32 | 32 (R) | 128 (R) |
| 8509 | 64 | 128 (R) | 128 (R) |
| 10324 | 64 | 32 (R) | 64 (R) |
| 17800 | 32 | > 256 (R) | 128 (R) |
| 17849 | 32 | > 256 (R) | 128 (R) |
| 18474 | 64 | > 256 (R) | 256 (R) |
| 18477 | 32 | > 256 (R) | 256 (R) |
| 18555 | 32 | > 256 (R) | 128 (R) |
| 19604 | 32 | > 256 (R) | > 256 (R) |

S: strain categorized as sensitive to the antibiotic based on CLSI MIC interpretative breakpoints.

R: strain categorized as resistant to the antibiotic based on CLSI MIC interpretative breakpoints.


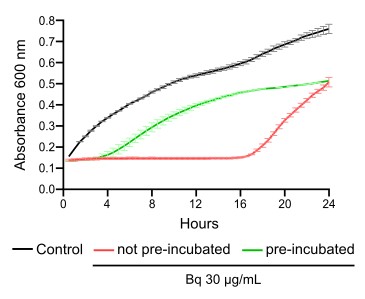


**Figure S1:** The antimicrobial activity of the blue 1,4-benzoquinone is affected by pre-incubation in MH broth. Growth kinetics of the *A. baumannii* 17978 strain were evaluated in MH broth containing 30 µg mL^-1^ of benzoquinone, added either at the start of the experiment (not pre-incubated) or 12 hours earlier (pre-incubated). Cultures without benzoquinone were assessed as controls. Data represents the average of three independent experiments done in triplicate; error bars represent standard error of the mean.


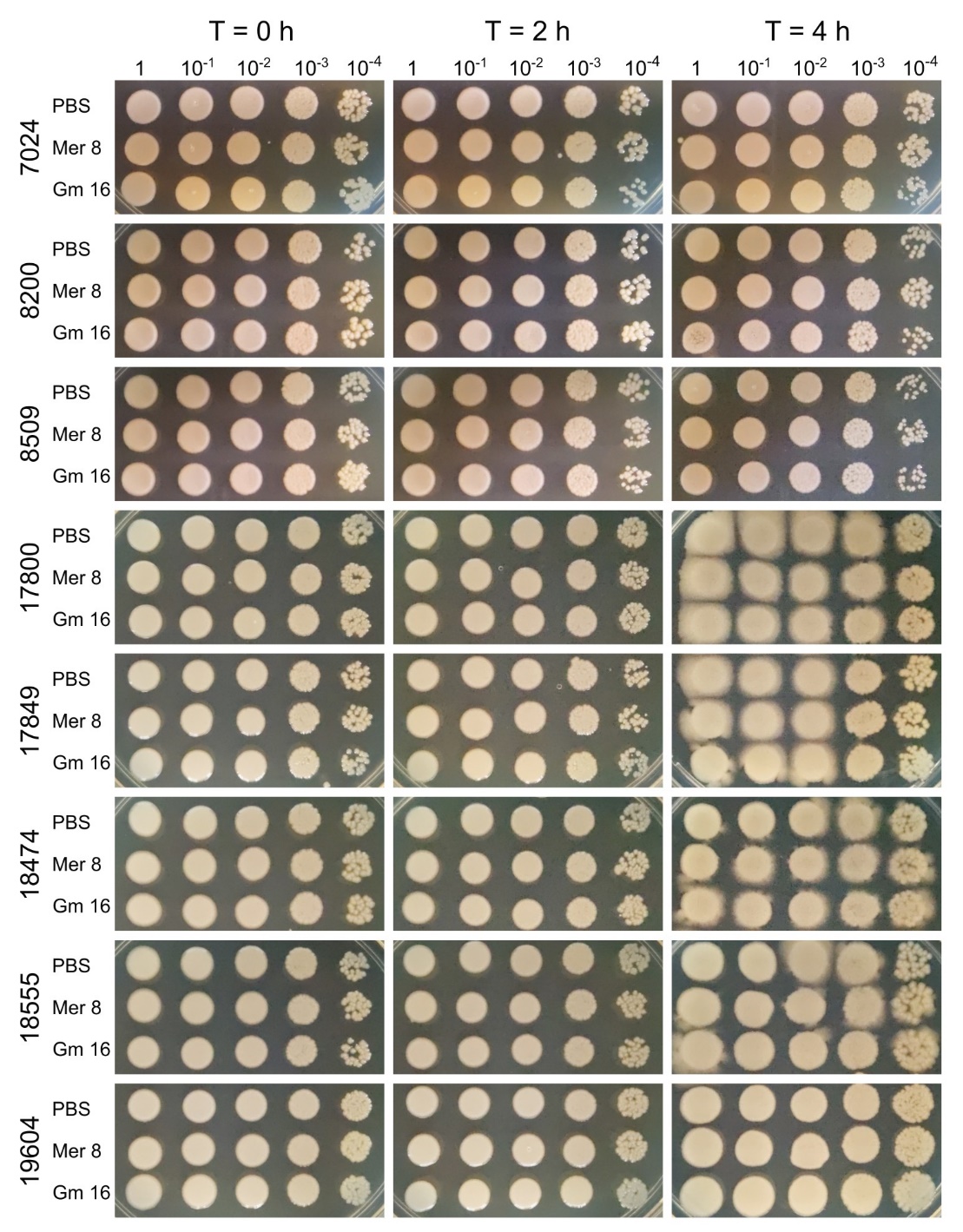


**Figure S2:** Effect of the antibiotics meropenem and gentamicin on the viability of MDR strains of *A. baumannii*. Bacterial suspensions in PBS were exposed to meropenem at 8 µg mL^-1^ (Mer 8) or gentamicin at 16 µg mL^-1^ (Gm 16) during 4 h with incubation at 37 °C. Bacterial suspensions not exposed to antibiotics are indicated as PBS. CFUs were recorded by spotting serially diluted samples of the bacterial suspensions on MH agar plates at different exposition times (0, 2, and 4 h). Representative images from independent experiments are shown.


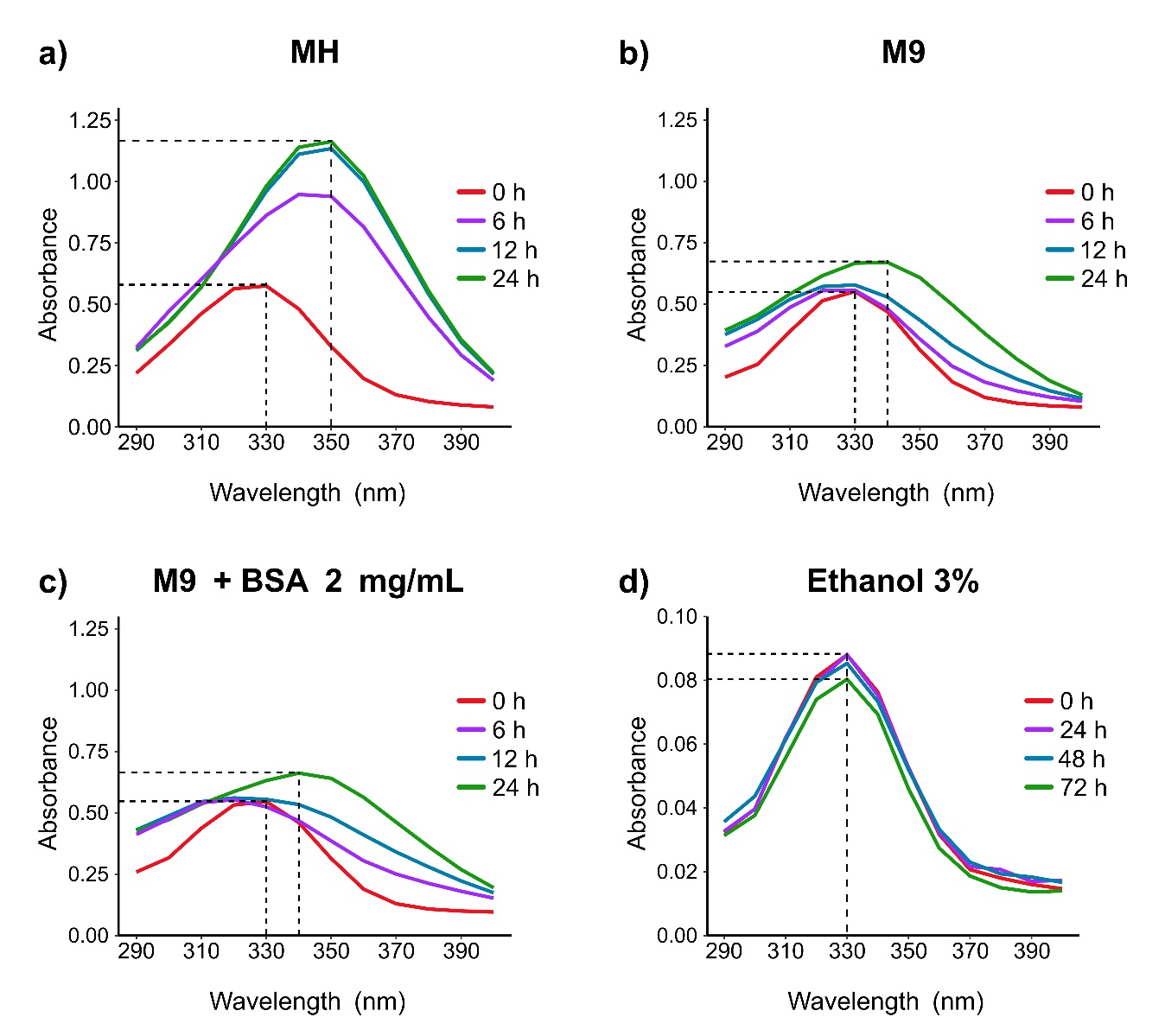


**Figure S3:** Analysis of the benzoquinone´s absorption spectra in different conditions without bacteria. The absorption spectra of the benzoquinone at 30 µg mL^-1^ was determined in: **a)** MH broth, **b)** M9 broth, **c)** M9 broth containing 2 mg mL^-1^ BSA, and **d)** 3% ethanol solution. Absorption spectra were obtained every hour during 24 h for the assays in culture media and every 24 h during 72 h for the assays in ethanol solution. Graphs show absorption spectra for selected times obtained from the averages of triplicates. Dotted lines indicate the absorbance and wavelength of the maximum absorption peak obtained at the first and last time tested.


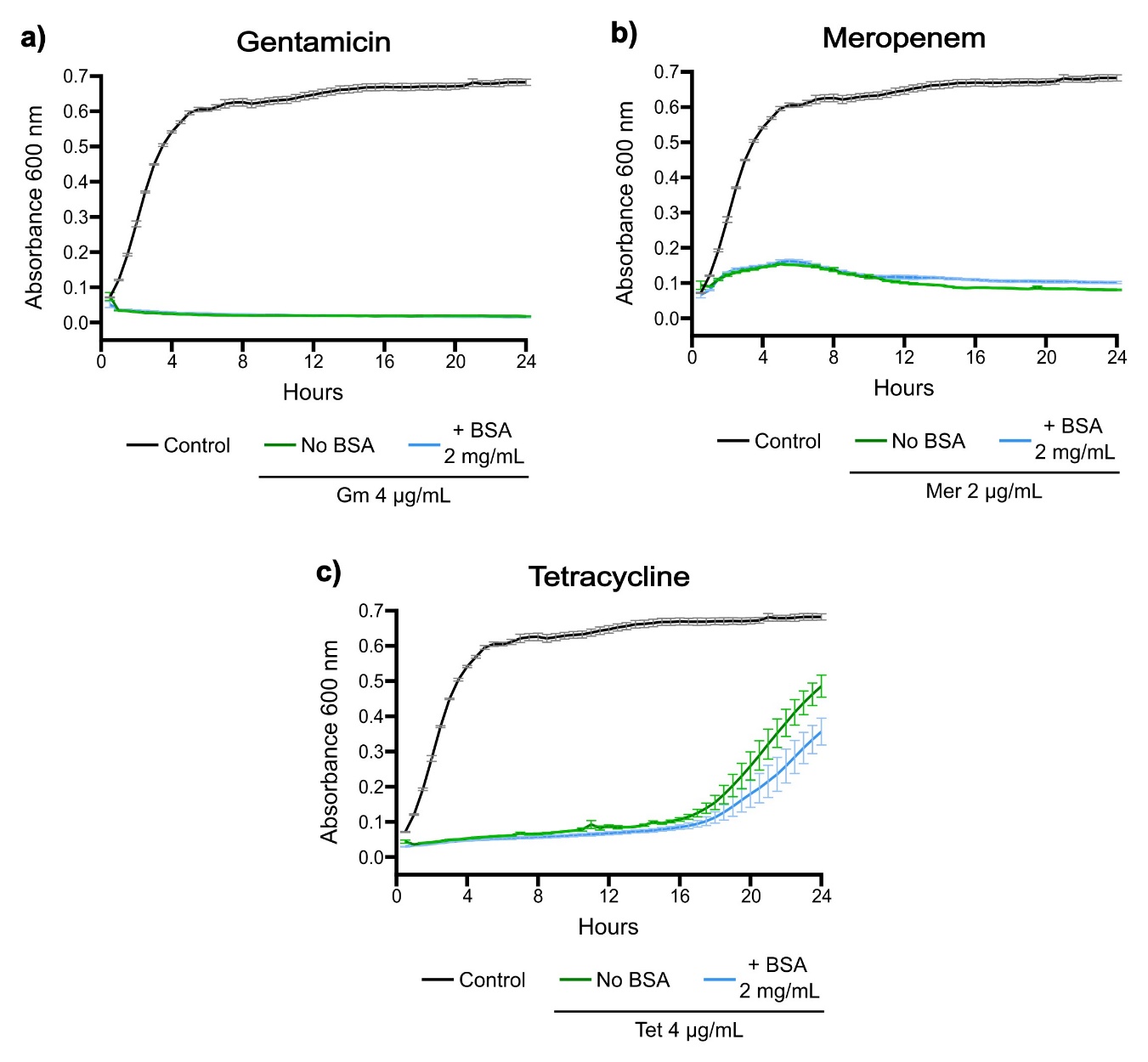


**Figure S4:** Antimicrobial activity of the antibiotics gentamicin, meropenem, and tetracycline in the presence of bovine serum albumin (BSA). Growth kinetics of the *A. baumannii* 17978 strain in M9 broth containing or not BSA at 2 mg mL^-1^, and containing the antibiotics: **a)** gentamicin at 4 μg mL^-1^, **b)** meropenem at 2 μg mL^-1^, or **c)** tetracycline at 4 μg mL^-1^. Control lacks antibiotics and BSA. Data represents the average of three independent experiments done in triplicate; error bars represent standard error of the mean.
